# The Cuyahoga Is Still Burning

**DOI:** 10.1289/ehp.11419

**Published:** 2008-04

**Authors:** Ellen K. Silbergeld, Jay P. Graham

**Affiliations:** Department of Environmental Health Sciences, Johns Hopkins Bloomberg School of Public Health, Baltimore, Maryland, E-mail: jgraham@jhsph.edu; esilberg@jhsph.edu

Almost 40 years ago, in 1969, the Cuyahoga River caught fire as it flowed through Akron and Cleveland, Ohio. Although it was not the only river to burn, the image of this burning river—the terrible wronging of nature—became a symbol of the urgency for a transformation of environmental policy for the environmental movement of the 1970s, driving the creation of the U.S. Environmental Protection Agency (EPA) and the rapid passage of major new environmental laws and regulations.

The Cuyahoga River doesn’t catch on fire any more, but somewhere along the way, we forgot something important about the environment and health. We forgot about the pathogens. We weren’t the only ones: In 1978 the World Health Organization predicted that by the year 2000 infectious diseases would no longer pose a threat to public health, even in the poorest countries. That was before HIV (human immunodeficiency virus), SARS (severe acute respiratory syndrome), MRSA (methicillin-resistant *Staphylococcus aureus*), and the return of highly pathogenic avian influenza (Morse 1993). To paraphrase lyrics from Billy Joel’s “We Didn’t Start The Fire” (Joel 1989), we have tried to stop the burning (and succeeded), but the pathogens keep on flowing.

Although the Cuyahoga River doesn’t burn, it is still in critical condition, filled with viruses, bacteria, and microparasites [U.S. Geological Survey (USGS) 2004]. The fish are back, and the people are back—fishing, swimming, and boating in the Cuyahoga Valley National Park, just as they are in Baltimore’s Inner Harbor (Baltimore, MD), the New York Bight, and many other rivers and lakes once devoid of aquatic life and human laughter. However, they are not safe. In the Cuyahoga, the USGS (2004) found *Salmonella*, *Clostridium*, enteroviruses, hepatitis A virus, *Cryptosporidium*, and *Giardia*. These are among the most common causes of infectious disease in the United States. In the rivers and streams flowing through Baltimore to the Inner Harbor of the Chesapeake Bay, researchers at Johns Hopkins University have found the same dangerous brew of disease-causing organisms. We carried out a study of urban anglers—people who fish in the urban watershed of Baltimore—and reported that, although some fish have elevated levels of polychlorinated biphenyls or mercury, anglers are at immediate risk of infection by *Cryptosporidium* merely by handling caught fish and crabs (Graczyk et al. 2007; Roberts et al. 2007).

The modern environmental movement began with a long overdue call to action concerning pesticides and other industrial chemicals (Hays 2000). Although levels of most persistent pesticides in humans and wild animals in the United States have fallen considerably since 1969, and exposures to lead in U.S. children have fallen by > 90% since 1976, that work is not finished. We have an urgent need to bring the pathogens back into the portfolio of environmental health research. Pathogens are increasing in virulence and in prevalence in the environment because of the inexorable collapse of unrepaired sanitary infrastructure, overloading of wastewater management due to suburban sprawl, and lack of attention to nonpoint inputs, particularly from industrial-scale food animal production. Agriculture is also a significant source of antibiotic-resistant pathogens, which are associated with the use of antibiotics as feed additives for the billions of chickens, swine, and cattle produced annually in the United States (Sapkota et al. 2007). Unfortunately, regulatory attention is intermittent; the U.S. EPA’s most recent regulations on controlling animal wastes do not include pathogens, and a recent congressional authorization for grants to municipalities and states is limited to controlling storm-water–related overflows from wastewater treatment plants. Pathogens are not even included in the fish advisories used by states, under the Clean Water Act (1977), to protect the health of recreational anglers. In fact, the way pathogen contamination is measured in surface waters has not been updated since the 1960s, even though we know that testing for so-called coliform bacteria provides little reliable information on viruses or microparasites, which are much more dangerous pathogens (National Research Council 2004).

These are environmental health issues of the highest priority. The National Institute of Environmental Health Sciences has a critical role in improving our knowledge on the role of the environment in the emergence and dispersion of infectious pathogens and human disease, including ecologic cycling of pathogens, the regulation of reservoirs of antimicrobial resistance, and the persistence of pathogens in soils and other media.

## Figures and Tables

**Figure:**
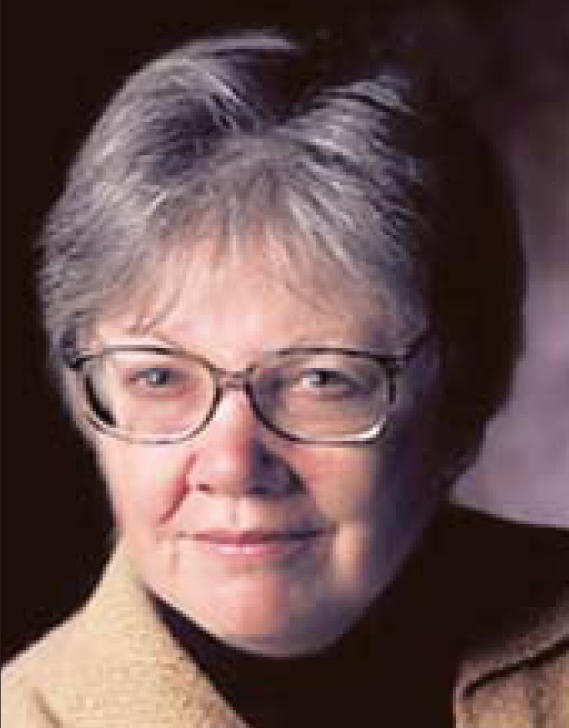
Ellen K. Silbergeld

**Figure:**
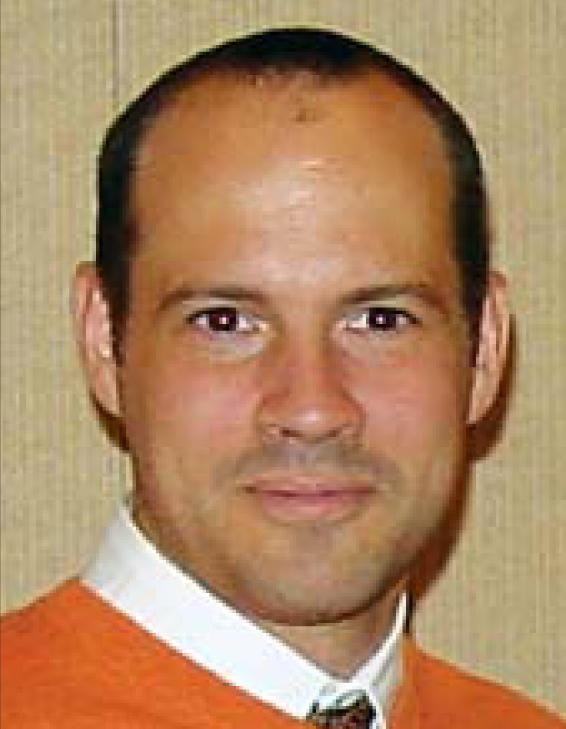
Jay P. Graham
